# P02-033 - CAPS diagnosis and treatment in an Israeli family

**DOI:** 10.1186/1546-0096-11-S1-A140

**Published:** 2013-11-08

**Authors:** Y Shinar, G Breuer, A Livneh, P Hashkes

**Affiliations:** 1 Heller Institute of Medical Research, Sheba Medical Center, Tel Hashomer, Israel; 2 Rheumatology Unit, Shaare Zedek Medical Center, Israel; 3 Hebrew University School of Medicine, Hebrew University, Jerusalem, Israel; 4 Sackler School of Medicine, Tel Aviv University, Tel Aviv, Israel; 5 Pediatric Rheumatology Unit, Shaare Zedek Medical Center, Jerusalem, Israel

## Introduction

Only one family in Israel, from Ethiopian Jewish origin has been diagnosed with the familial cold autoinflammatory syndrome phenotype of the cryopyrin associated periodic syndromes (CAPS)[1].

## Case report

We confirmed the Muckle-Wells syndrome phenotype of CAPS by *NLRP3* genetic testing in a three generation family of Turkish Jewish origin, previously diagnosed with familial Behcet disease due to the presence of mucosal ulcers in several family members with the finding of the HLA-B51 antigen in at least one family member. Eight family members including a deceased grandfather, 4 of his daughters and three grandchildren had brief episodes of fever and chills, accompanied by headache, myalgia, arthralgia, and an urticarial skin rash. Most family members had substantial hearing loss. None developed amyloidosis. Four family members tested for a *NLRP3* pathogenic variant had the known NM_001243313.1:c.1043C>T, p.Thr348Met variant[2]. Following initiation of treatment with canakinumab (150 mg every 8 weeks) and colchicine for mucosal ulcers all disease symptoms resolved and acute phase reactants normalized except for persistent headaches in one grandchild and tinitus in another. The health-related quality of life of the treated grandchildren markedly improved.

## Discussion

*NLRP3* genetic testing was instrumental in the diagnosis of CAPS in this family, particularly as some family members presented with atypical features suggestive of Behcet disease, which is much more common in Israel. Although CAPS is a rare disease, additional cases with other *NLRP3* variants may exist in Israel.

## Disclosure of interest

Y. Shinar: None Declared, G. Breuer: None Declared, A. Livneh Grant / Research Support from: Novartis, P. Hashkes Grant / Research Support from: Novartis, Consultant for: Novartis, Speaker bureau of: Novartis.
